# Multi-Omics Study of Keystone Species in a Cystic Fibrosis Microbiome

**DOI:** 10.3390/ijms222112050

**Published:** 2021-11-07

**Authors:** Cynthia B. Silveira, Ana G. Cobián-Güemes, Carla Uranga, Jonathon L. Baker, Anna Edlund, Forest Rohwer, Douglas Conrad

**Affiliations:** 1 Department of Biology, University of Miami, 1303 Memorial Dr., Coral Gables, FL 33146, USA; 2 Department of Biology, San Diego State University, 5500 Campanile Dr., San Diego, CA 92182, USA; ana.naiboc@gmail.com (A.G.C.-G.); frohwer@gmail.com (F.R.); 3 Viral Information Institute, San Diego State University, 5500 Campanile Dr., San Diego, CA 92182, USA; 4 Genomic Medicine Group, J. Craig Venter Institute, San Diego, CA 92037, USA; curanga@jcvi.org (C.U.); jobaker@jcvi.org (J.L.B.); aedlund@jcvi.org (A.E.); 5 Department of Pediatrics, University of California San Diego School of Medicine, San Diego, CA 92037, USA; 6 Division of Pulmonary, Critical Care and Sleep Medicine, University of California San Diego, San Diego, CA 92037, USA; dconrad@ucsd.edu

**Keywords:** clindamycin, anaerobes, fermentation, mucus plugs, WinCF, metagenomics, metabolomics, transcriptomics

## Abstract

Ecological networking and in vitro studies predict that anaerobic, mucus-degrading bacteria are keystone species in cystic fibrosis (CF) microbiomes. The metabolic byproducts from these bacteria facilitate the colonization and growth of CF pathogens like *Pseudomonas aeruginosa*. Here, a multi-omics study informed the control of putative anaerobic keystone species during a transition in antibiotic therapy of a CF patient. A quantitative metagenomics approach combining sequence data with epifluorescence microscopy showed that during periods of rapid lung function loss, the patient’s lung microbiome was dominated by the anaerobic, mucus-degrading bacteria belonging to *Streptococcus*, *Veillonella*, and *Prevotella* genera. Untargeted metabolomics and community cultures identified high rates of fermentation in these sputa, with the accumulation of lactic acid, citric acid, and acetic acid. *P. aeruginosa* utilized these fermentation products for growth, as indicated by quantitative transcriptomics data. Transcription levels of *P. aeruginosa* genes for the utilization of fermentation products were proportional to the abundance of anaerobic bacteria. Clindamycin therapy targeting Gram-positive anaerobes rapidly suppressed anaerobic bacteria and the accumulation of fermentation products. Clindamycin also lowered the abundance and transcription of *P. aeruginosa,* even though this patient’s strain was resistant to this antibiotic. The treatment stabilized the patient’s lung function and improved respiratory health for two months, lengthening by a factor of four the between-hospitalization time for this patient. Killing anaerobes indirectly limited the growth of *P. aeruginosa* by disrupting the cross-feeding of fermentation products. This case study supports the hypothesis that facultative anaerobes operated as keystone species in this CF microbiome. Personalized multi-omics may become a viable approach for routine clinical diagnostics in the future, providing critical information to inform treatment decisions.

## 1. Introduction

Keystone species are members of a community with disproportionately high importance to community stability despite being less abundant than other community members [1]. In the human microbiome, this ecological concept gave rise to the keystone-pathogen hypothesis, positing that “certain low-abundance microbial pathogens can orchestrate inflammatory disease by remodeling a normally benign microbiota into a dysbiotic one” [2]. However, microbial ecology studies identify keystone species mainly based on their central position in network analyses [3,4]. One caveat of this approach is that these networks are often built based on co-occurrence relationships in metagenomes and transcriptomes [5,6]. Identifying real keystone species requires manipulating the microbiome in vivo [7,8].

The fast pace of technological advancements for the description of human microbiomes during health and disease is propelling the field towards informed manipulation of the microbiome. Many strategies have been proposed to steer dysbiotic microbial communities towards healthy states, including probiotics, prebiotics, phage therapy, and microbiome transplants [9]. In reality, manipulation of the microbiome has been performed for decades through the use of narrow-spectrum antibiotics during clinical treatments [7,8]. Multiple omics studies have characterized the microbiome changes resulting from antimicrobial drugs [10]. However, to the best of our knowledge, only one study applied omics tools to prospectively inform the choice of antibiotics [11]. In this study, the abundance of the pathogen *P. aeruginosa* decreased in both patient groups, receiving only standard care receiving additional individualized antibiotics targeting the third and fourth most abundant members of the community, according to 16S sequencing. However, the microbiome community composition returned to initial state after one month. A combination of multi-omics approaches may be a viable (yet still costly) solution for guiding the treatment of polymicrobial diseases characterized by highly individualized microbial communities in cases where standard antibiotic treatment does not improve patient’s health [12,13,14]. One of these approaches is the cystic fibrosis rapid response (CFRR), where metagenomes, metatranscriptomes, metabolomes, and viromes are generated within 48 h from patients undergoing rapid loss in lung function [12]. The CFRR has illuminated the causes of lung function loss that could not be solved by standard clinical diagnosis and treatment. Yet, to date, none of the CFRR studies has timely informed a successful choice of treatment. 

Cystic fibrosis (CF) patients suffer from chronic polymicrobial infections throughout their lives, being treated with multiple antibiotics from an early age [15]. Alterations in anion transport and mucociliary clearance of the respiratory epithelium facilitate the microbial colonization [16,17,18,19]. These communities elicit strong innate immune responses that drive the airway wall remodeling and gas exchange abnormalities, eventually resulting in respiratory failure or a need for lung transplantation in over 80% of CF patients. The climax–attack ecological model [20] builds upon the polymicrobial nature of CF airway infections [20,21,22,23,24,25]. The attack community consists of transient members that elicit strong innate immune responses and acute deterioration [26,27]. In contrast, climax communities cause chronic infections. They include most of the classic CF pathogens, such as *P. aeruginosa,* which are inherently resistant to antimicrobial therapy, grow slowly forming biofilms, and generate suboxic environments in mucus plugs [28,29,30]. Hypoxic metabolisms are frequent in CF patients with advanced lung disease or during exacerbations. In these periods, microbes use alternative electron acceptors (nitrates, sulfates, iron, and fumarate) and fermentation [27,31,32], accumulating toxic metabolic products in the lung [33,34]. 

Previous network analysis and in vitro studies suggested that anaerobic bacteria function as keystone species, sustaining the microbial community growth and disease progression in CF [35,36]. The mechanistic basis of this hypothesis is the cross-feeding that occurs as mucin-degrading anaerobes release fermentation products that sustain the growth of pathogens such as *P. aeruginosa*, an inefficient mucus-degrading bacterium [36]. Amino-acid incorporation experiments demonstrate that anaerobes are active in the lung communities even at low abundances [37]. The minimum inhibitory concentration of the most susceptible member of a community grown under this nutritional dependency defines the inhibitory concentration of the whole community [38]. These data suggest that clinically targeting the weakest link in the nutritional consortium could control polymicrobial infections. However, clinical studies showed that patients treated with antibiotics with broad antibiotic activity against anaerobes showed no difference in lung function recovery compared with those treated with antibiotics with minimal anaerobe coverage [39]. Here, a multi-omics-informed clinical treatment using clindamycin to target Gram-positive anaerobes offered an invaluable opportunity to test *in vivo* the CF keystone hypothesis. Quantitative multi-omics and community culturing showed that clindamycin suppressed not only anaerobes but also *P. aeruginosa.* Disturbance of microbial cross-feeding was observed through transcriptomics and improved the patient’s health by extending the between-hospitalization time by a factor of four. This study demonstrates that standard narrow-spectrum antibiotics combined with personalized multi-omics represent a low-risk manipulation tool to test hypotheses and guide future approaches for broadening targeted microbiome manipulation.

## 2. Results

Clinical data: The CF Rapid Response (CFRR), a real-time multi-omics study of a CF patient during a period of rapid loss in lung function, was launched at the time of an exacerbation event of CF146 (33). At Day 0 (First day of CFRR), the patient displayed an acute decline in respiratory health characterized by severe cough, dyspnea, and a drop in his percent predicted Forced Expiratory Volume in 1 s (ppFEV1) to 44%, 9% below the median ppFEV1 (Figure S1). In the 27 days of hospitalization that followed (period A in this study), the patient received colistin, vancomycin, piperacillin-tazobactam, and ceftazidime-avibactam. The ppFEV1 increased to 59%, leading to the patient’s discharge. During the following eight days (period B), the patient was off antibiotics. Six days after hospital discharge, the ppFEV1 rapidly decreased to 50%, accompanied of intense cough and shortness of breath. In period C, oral clindamycin treatment was initiated, improving overall symptoms, including cough and shortness of breath, and maintaining ppFEV1 at 49%.

**Figure 1:**
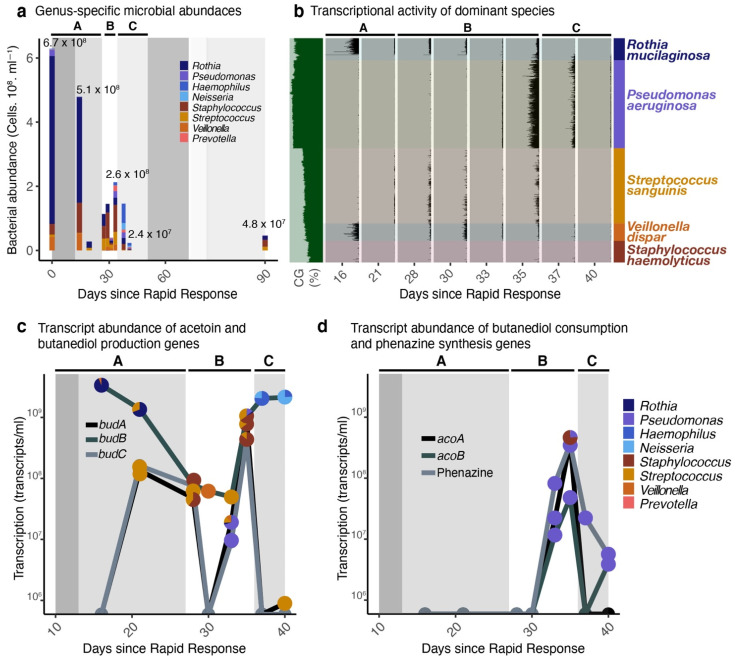
Bacterial abundances and transcriptional activity. (**a**) Changes in abundance of most abundant bacterial genera in the sputum from patient CF146. Abundances (in cell counts per mL of sputum) were obtained as the product of the fractional abundance from metagenomes normalized by genome size and by the total cell counts from epifluorescence microscopy. (**b**) Recruitment of metatranscriptomic reads to contigs obtained from sequencing the genomes of clinical bacterial isolates. The height of each peak in the plot denotes mean coverage of a contig divided by overall sample mean coverage and scaled to the microbial abundances per mL of sputum sample. As these are draft and not complete genomes, the position of each contig along the y axis is defined by k-mer similarity (k = 4) rather than position in the genome. The letters A, B and C within the plot area indicate the different antibiotic treatment periods. (**c**) Abundance of transcripts involved in the pathway of acetoin and butanediol production (*budA*, *budB*, and *budC*). Each point in the plot is divided by the taxonomic assignment of the transcripts. The transcript abundance per ml (y axis) was calculated as the product of the number of transcripts per Kb per taxon and the total number of Kbs of a that same taxon, calculated from the abundances and genome sizes. (**d**) Abundance of transcripts involved in the pathway of acetoin and butanediol consumption (*acoA* and *acoB*) and phenazine production (*phzABCDEFG*, shown as a sum of all transcripts in this gene cluster). Each point in the plot is divided by the taxonomic assignment of the transcripts. The transcript abundance per mL (y axis) was calculated as in 4B.

Bacterial abundances: The bacterial load in the sputum sample collected at Day 1 was 6.7 × 10^8^ cells per ml, and *R. mucilaginosa* dominated the community (78% of the community, Figure 1). After two weeks of intravenous antibiotics in Period A, the bacterial load decreased to 2.9 × 10^7^. Most members of the community decreased their abundance, except for *Staphylococcus haemolyticus* and *Veillonella dispar*. In period B, when the patient was off antibiotics, the microbial abundance increased to 2.6 × 10^8^. At this point, *S. haemolyticus* and *S. sanguinis* were the most abundant bacteria, followed by *R. mucilaginosa* and *P. aeruginosa*. The clindamycin treatment (Period C) decreased microbial load to 2.4 × 10^7^, and the community became dominated by *Haemophilus* sp. and *Neisseria* sp. Both *Haemophilus* sp. and *Neisseria* sp. decreased in abundance to less than 0.001% of the community after two months.

Bacterial transcriptional activity: Transcriptome mapping to the draft genome sequences of *Rothia mucilaginosa*, *Streptococcus sanguinis*, *Staphylococcus haemolyticus*, *Veillonela dispar*, and *Pseudomonas aeruginosa* isolated from the patient’s sputum showed the community activity. The most active community members were *R. mucilaginosa*, *S. sanguinis*, *V. dispar*, and *P. aeruginosa*, respectively (Figure 1b). *S. haemolyticus* had low transcriptional activity, despite its 54% abundance in the metagenome three days after hospital discharge. During intravenous colistin treatment, *R. mucilaginosa* was the most transcriptionally active community member. Upon release from antibiotics, *V. dispar* transcripts increased, followed by *S. sanguinis* and *R. mucilaginosa*. After six days off antibiotics, *P. aeruginosa* became the most active member of the community. Oral clindamycin treatment correlated with a decrease in transcript abundance not only in the target species *S. sanguinis*, but also in *V. dispar*, *R. mucilaginosa*, and *P. aeruginosa*.

Acetoin metabolism genes are responsible for the synthesis of the 2,3-butenediol and butanedione, critical metabolites in the cross-feeding between *Streptococcus* spp. and *P. aeruginosa* [35]. The genes encoding acetoin biosynthesis acetolactate synthase (*budB*), acetolactate decarboxylase (*budA*), and butanediol dehydrogenase (*budC*) were expressed by *Streptococcus* spp. and *Staphylococcus* spp. at higher levels in periods of lung function loss (Figure 1c). *R. mucilaginosa* also expressed *budB* early in the sampling period, and *Haemophilus* sp. and *Neisseria* sp. expressed these genes after the onset of clindamycin treatment. *P. aeruginosa* was the taxon with the highest expression of the genes *acoA* and *acoB*, whose products catabolize acetoin for utilization in central metabolism (Figure 1d). The production of phenazine followed that of *aco* genes by *P. aeruginosa* (Figure 1d). Phenazines are virulence factors that act as alternative electron acceptors allowing *P. aeruginosa* to thrive in anoxic regions of biofilms and mucus plugs.

**Figure 2:**
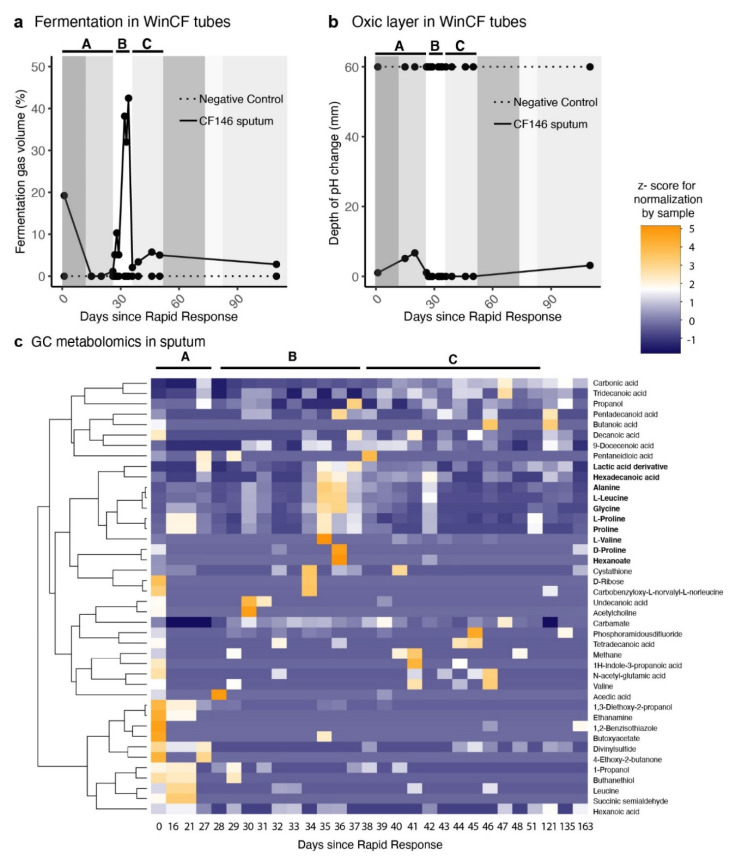
Fermentation in the sputum of patient CF146. (**a**) production of fermentation gas in in WinCF capillary tubes inoculated with sputum microbial communities in artificial sputum media; (**b**) depth of pH change in WinCF capillary tubes. The depth of tube where the pH changes corresponds to the transition from the oxic to anoxic environment and is analogous to the oxygen penetrance in the mucus plugs in the lung [34]. (**c**) Relative abundances of polar molecules identified by GC-MS metabolomic analysis. Abundances were normalized by rows (days) to allow between-sample comparisons and plotted are the z-scores of this normalization. The letters A, B and C within the plot area in the three panels indicate the different antibiotic treatment periods.

Fermentation and metabolomics: On Day 1, the community cultures in the Winogradsky CF (WinCF) system produced fermentation gas that occupied 19% of the WinCF tube (Figure 2a). Simultaneously, the pH decreased below five starting at 0.33 mm of the tube (Figure 2b). The pH change depth corresponds to the oxic/anoxic transition and is analogous to the oxygen penetrance in the mucus plugs in the lung [34]. The amount of fermentation gas decreased to non-detectable after two weeks of antibiotic treatment in period A (Figure 2a), and the depth of pH change in the tubes increased (Figure 2b). After the hospital discharge, gas production increased to 42%. This period corresponded to a decrease in pH change depth to 0 mm, loss of lung function, and overall deterioration of patient’s health. The clindamycin treatment was initiated nine days after the hospital discharge. Clindamycin treatment correlated with a sustained decrease in the fermentation levels after 24 h, which remained below 3% for 60 days. The GC-MS metabolomic profiles showed that during the period the patient was off antibiotics, there was an increase in the abundance of fermentation products (Figure 2c). Lactic acid peaked 6–8 days after the patient stopped intravenous colistin treatment and coincided with the increase in fermentation gas and deterioration of heath. There was also an increase in the abundance of free amino acids.

**Figure 3:**
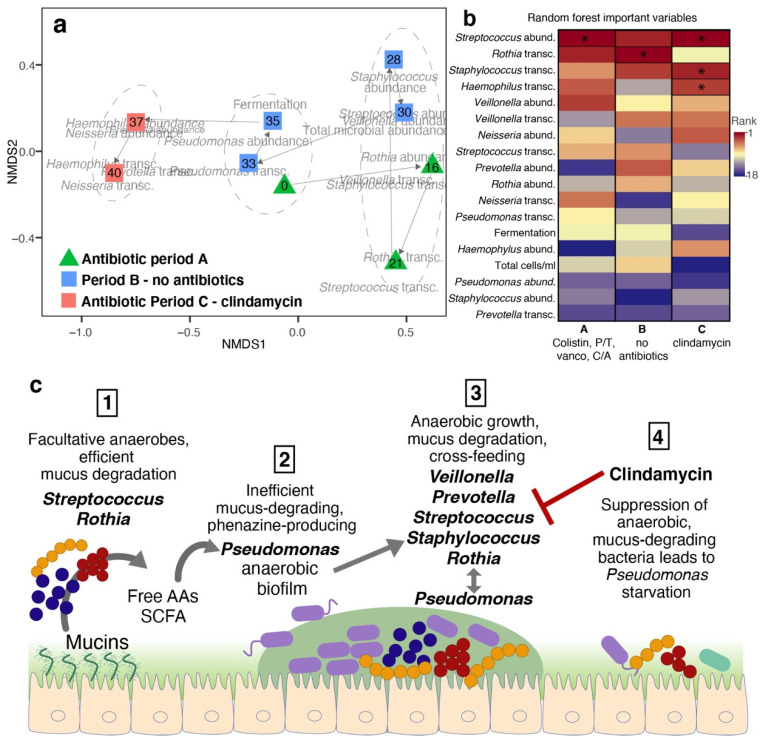
Microbial community succession. (**a**) Non-metric multidimensional scaling (NMDS) of sputum samples over time. The variables used as input for the NMDS were fermentation, total microbial abundances, genera-specific microbial abundances, and genera-specific transcriptomic activity. Symbols are color-coded by antibiotic treatment periods (green: A—colistin, vancomycin, ceftazidime-avibactam, and piperacillin-tazobactam; blue: B—no antibiotics; pink: C—clindamycin), and numbers inside the symbols indicate days since hospital admission event when Rapid Response was initiated. The dotted ellipses indicate health status groups supported by an unsupervised random forest analysis followed by clustering using Ward distances (out of bag error = 11.1%). (**b**) Important variables differentiating treatment periods in a classification random forest analysis supervised by treatments. The variables marked by an asterisk and surrounded by a black box indicate variables with *p*-values less than 0.05 in the permutational test. (**c**) Conceptual model of succession events in patient CF146: (1) The mucosal surfaces are colonized by facultative anaerobes, including *Streptococcus* sp. and *Rothia* sp., capable of efficient mucus degradation producing free-amino acids and short-chain fatty acids (SCFA, i.e., propionate, acetate, butyrate, and butanediol). (2) The free amino acids and SCFAs open a niche for the growth of *P. aeruginosa*, which degrades mucins poorly. *P. aeruginosa* grows forming an anaerobic biofilm and produces phenazines. (3) The *P. aeruginosa* biofilm facilitates the growth of obligate anaerobes such as *Veillonella* sp. and *Prevotella* sp. There is an overall growth of the whole microbial community, with *Pseudomonas* benefitting from the metabolic products from anaerobes. (4) The onset of clindamycin treatment suppresses the growth of Gram-positive anaerobes, such as *Streptococcus* sp. and *Staphylococcus* sp. The suppression of mucus-degrading bacteria removes the main nutritional source for *P. aeruginosa*, leading to an overall decrease in microbial abundances. Period A corresponds to 28 days of hospitalization when the patient was treatment with colistin, vancomycin, piperacillin-tazobactam, and ceftazidime-avibactam; in period Period B the patient was released and was off antibiotics; Period C is the clindamycin treatment.

Microbial community structure: Figure 3a shows a 2D projection of sample clusters identified by an unsupervised random forest analysis of microbial abundances, transcriptional activities, and community cultures (NMDS, stress = 0.05, Linear fit R^2^ = 0.98). The first cluster includes samples from periods of lowest ppFEV1 and worst symptoms, coinciding with the highest fermentation levels and highest *P. aeruginosa* abundance and transcriptional activity. The antibiotic treatment in period A lowered microbial load and correlated with the abundance and transcriptional activity of *Rothia mucilaginosa.* In period B, the abundance of *Streptococcus sanguinis*, *Staphylococcus haemolyticus*, and *Veillonella dispar* increased as the patient’s health deteriorated. A return to low lung function and high fermentation followed, coinciding with high *P. aeruginosa* abundance and transcriptional activity. 

Clindamycin treatment in period C drove the community to a third state, characterized by high *Neisseria* sp. and *Haemophilus* sp. abundance and transcriptional activity, with low total microbial load and fermentation. The abundance of *S. sanguinis* was the most important variable differentiating the three clusters, followed by the abundance of *P. aeruginosa* and *S*. *sanguinis* transcriptional activities. A random forest analysis supervised by the antibiotic regimes (Periods A, B, and C) showed an out of bag error rate estimate of 11.1%. *S*. *sanguinis* abundance was the variable with the highest importance differentiating the periods, followed by *R. mucilaginosa, S. haemolyticus, and Haemophilus* sp. transcriptional activities (Figure 3b, *p*-values in the permutational random forest test were 0.002, 0.009, 0.02, and 0.04, respectively).

## 3. Discussion

Microbial colonization of the lungs in CF patients represents a case of complex microbial dysbiosis driving disease progression, often without the identification of an individual causative pathogen [30]. Metabolic networks based on co-occurrence have predicted that *Streptoccoccus*, *Prevotella*, and *Veillonella* are the genera with the highest *keystoneness* in CF [35]. These species are efficient mucus-degraders, and their anaerobic metabolism releases short-chain fatty acids that sustain the growth of *P. aeruginosa* [36]. Our community-culture and multi-omics data show that these pathways were active in the lungs of CF146. Based on the multivariate statistical analysis of the combined multi-omics dataset, we propose the following model of community succession facilitated by anaerobes in this CF microbiome (Figure 3c). First, facultative anaerobes, including *Streptococcus* spp. and *R. mucilaginosa*, efficiently degraded mucins, producing free-amino acids and short-chain fatty acids (SCFA, i.e., propionate, acetate, butyrate, and butanediol). The free amino acids and SCFAs open a niche for the growth of *P. aeruginosa*, which degrades mucins poorly. *P. aeruginosa* forms an anaerobic biofilm and produces phenazines. Volatile fermentation products such as lactic acid and 2,3-butanediol have been previously detected in CF breath gas [34,40]. These molecules can induce pyocyanine production, dormancy, and biofilm formation in *P. aeruginosa*, stimulating its growth and virulence [41,42]. Moreover, 2,3-butanediol has a direct toxic effect on human cells [34]. We further propose that the anoxic biofilm created by *P. aeruginosa* facilitates the growth of strict anaerobes, including *Veillonella* spp. and *Prevotella* spp., fueling a positive feedback loop that sustains the growth of the whole microbial community. The onset of clindamycin treatment suppresses mucus-degrading anaerobes, removing the primary nutritional sources for *P. aeruginosa*, and destabilizing the community.

Clindamycin is a semisynthetic derivative of lincomycin acting on ribosome translocation that inhibits protein synthesis. It affects Gram-positive anaerobes, such as *Staphylococcus* spp. and *Streptococcus* spp., and Gram-negative anaerobes, including *Prevotella* spp. [43]. In addition to these anaerobes, clindamycin reduced the abundance and activity of *P. aeruginosa*, which is resistant to this drug (Figure 1). The decrease in *P. aeruginosa* indicates its dependency on *Streptococcus* spp. and *Veillonella* spp. byproducts for growth. *P. aeruginosa*’s large genome is highly adapted to mucus plugs, encoding alternate oxidative metabolism and virulence factors [44]. However, *P. aeruginosa* grows poorly on mucins and depends on cross-feeding by mucus-fermenting anaerobes [36]. *In vitro* antibiotic challenge targeting a mucin-fermenting community containing *Veillonella parvula*, *Fusarium nucleatum*, *Prevotella melaninogenica*, and *Streptococcus parasanguinis* in co-cultures with *P. aeruginosa* controlled both the fermenters and *P. aeruginosa* [45]. These antibiotic challenges indicated that the total community minimum inhibitory concentration dropped to that of the weakest link—the least resistant species that provides resources to other community members [45]. The dataset presented here provides evidence that the weakest link phenomenon occurs *in vivo* and corroborates that *Streptococcus* and *Veillonella* operated as keystone genera in this CF microbiome. Clindamycin is not traditionally used to treat CF lung infections, and its long-term use can facilitate the development of colitis by *Clostridium difficile* colonization [46]. Likewise, colistin poses a risk of kidney toxicity, which precludes its long-term use [47]. The lack of antibiotic drugs that can stabilize the lung microbial community in the long-term demands the development of novel strategies to manipulate the CF microbiome by removing keystone species and metabolisms.

Expectorated sputum is subject to contamination by saliva and oropharynx flora during expectoration. However, 50–80% saliva contamination would be necessary to explain the abundances of *Rothia*, *Streptococcus,* and other common oral genera in the periods of lowest lung function shown here, which is unrealistic given the purulent sputum expectorated by the patient during the entire study period. Sputum samples mostly represent the communities coming from the lower respiratory tract [28,48], and anaerobes are active inside expectorated mucus plugs [38]. Differences between sputum and the lower tract may increase in end-stage patients because many of the airways undergo bronchiectasis and become clogged [49], which is not the case in this study. Therefore, saliva contamination cannot explain the patterns observed in the present dataset.

A major advancement of this study is the introduction of a quantitative metagenomics and metatranscriptomics approach combining sequence data with epifluorescence microscopy. The use of relative abundances in omics studies is considered one of the most significant bottlenecks in the microbiome field [50]. Internal standards have been applied to biological samples prior to sequencing to obtain absolute abundances [51,52]. A direct comparison between the approach introduced here and the use of internal stands was not performed here. Yet, epifluorescence microscopy is a standard and accurate method for the quantification of total bacterial abundances [53] and is not subject to biases in the efficiency of nucleic acid extraction that may interfere with quantifications [54]. Both methods provide a better assessment of the community composition and activity than simple relative abundances for between-sample comparisons.

The multi-omics case study presented here provides clinical support for the synergism between Gram-positive anaerobes and classic pathogens (i.e., *P. aeruginosa*) in CF. While the keystone anaerobe hypothesis has been probed in vitro and in correlation-based omics studies, a debate on the role of anaerobes persists in the CF field [37,38,39,55,56]. The clinical study presented here offers critical information to the cystic fibrosis research community and, more broadly, to those studying polymicrobial diseases, exposing the need for future clinical studies with large patient cohorts. Previous studies with larger sample sizes based on 16S rRNA data have shown that the low diversity and dominance of a single pathogen are indicative of rapid lung function loss [56]. This pattern is consistent with the concept of keystone species presented here. Community members with low abundance had disproportional importance in community function by nutritionally sustaining a dominant pathogen. While the cost of a multi-omics study as the one presented here is currently prohibitive for full clinical deployment, the WinCF is an available method to quantify fermentation, allowing the selection of patients for in-depth studies. Future studies with large patient cohorts will show whether the keystone dynamics observed here is common in cystic fibrosis microbiomes.

## 4. Methods

Clinical data: Sample collection procedures and access to clinical data were approved by the institutional review boards (IRBs) of the University of California San Diego (UCSD) (HRPP 081510), and San Diego State University (IRB approval number 1711018R). Clinical microbiology and spirometry tests were performed for clinical indications during the normal care of the patient. Spirometry tests were used to calculate the percentage of predicted FEV1 (forced expiratory volume in one second), as previously described [57]. The age*FEV1% predicted product is a derived variable calculated by multiplying the age of the individual at the most recent FEV1 by the best FEV1% during the previous year and is used to assess disease risk [58,59]. ppFEV1 is a standard metric to evaluate lung function, as the volume of exhaled air decreases when the lungs clog due to infection and inflammation. Patient 146 is a 33-year-old male (delF508/Q1382X) who has highly variable lung function as assessed by the dynamics of his percent predicted Forced Expiratory Volume in 1 s (ppFEV1) [59]. In the year preceding this study, the patient had ten periods of rapid lung function loss that led to hospitalization and intravenous (IV) antibiotic treatments (Figure S1). The ppFEV1 varied between 38% and 67% during that year (median = 53 ± 5.7 SD). The clinical microbiology tests were positive for *P. aeruginosa*; however, the patient’s lung function did not stabilize with oral antibiotic treatment after each hospital discharge.

WinCF community culture: Winogradsky CF community culture was modified from the work of Quinn et al. (2015) by adjusting the pH 7.4 prior to use in WinCF experiments [35,60,61,62]. Briefly, sputum samples were collected by expectoration, homogenized, and diluted 10-fold into sterile ASM. The mixture was inoculated into capillary tubes that were plugged at one end and then laid horizontally in a 100% humidity environment at 37 °C. At the end of incubations, capillary tubes were imaged in the dark on white backlight (5.5 × 9 inches^2^ light box, Logan Electric, Chicago, IL, USA) using a Canon EOS Rebel T3 camera (Canon USS Inc., Melville, NY, USA). All images were taken under identical settings (manual focus, ISO 3200, Aperture F4.5) and saved in raw and JPEG format. Gas production was quantified as the percentage of capillary tube length occupied by gas. The air penetrance in the capillary was defined as depth of the tube where pH dropped below 5, as indicated by color change in the phenol red/bromocresol purple tubes (pH drops due to fermentation products). Negative control tubes were filled with media and reagents but not inoculated with sputum and incubated alongside the treatment tubes. If the negative control tubes showed no signal of microbial growth (color change and gas production), the experiment was kept and described in this study, whereas if there was any signal of microbial growth, which indicated contamination, that experiment was discarded.

Metagenomes: Total DNA was extracted from sputum samples using an adapted PowerSoil DNA Isolation kit protocol (Qiagen, Hilden, Germany). The samples were subject to 5 freeze-thaw cycles (5 min flash-freeze, 5 min at 100 °C) followed by bead-beating for 45 min using the bead tubes from the PowerSoil kit. The DNA was eluted in molecular-grade water and metagenome libraries were constructed using a Nextera DNA library preparation kit (Illumina, San Diego, CA, USA). Libraries were sequenced on an Illumina MiSeq platform Reagent Kit v3 150-cycle (Illumina, San Diego, CA, USA) with depth of 1.5 to 3 M reads per sample. Negative controls were included in each batch of sample preparation from extraction to sequencing library quality control. If the negative controls generated zeros in the final step of quality control, that batch of samples was sequenced and included in the study, if not, the samples were discarded, and the procedure re-initiated from the raw biological sample. Quality filtering and dereplication were done using PRINSEQ++ [63] with minimum quality threshold 20, dereplication and entropy threshold 50. Cloning vector sequences were removed using SMALT [64] with 80% identity against the NCBI UniVec database. Human genome sequences were removed using SMALT with 80% identity against the human reference genome GRCh38 (Table S1, Supplementary Data S1). Microbial taxonomy assignments at the genus level were made from SMALT with 96% identity against the NCBI RefSeq database of complete bacterial genomes.

Bacterial isolation and genome sequencing: Fresh sputum sample from the first day of hospitalization in this study (Day 0) were resuspended in saline buffer and plated on BHI agar. Plates were incubated overnight at 37 °C. Colonies with distinct morphologies were picked and re-plated on BHI plates. Isolates had their 16S gene sequenced using the Sanger method and one representative of the each of the five most abundant species in the metagenomes (by sequence recruitment at 96% identity) were selected for full genome sequencing: *Rothia mucilaginosa*, *Streptococcus sanguinis*, *Staphylococcus haemolyticus*, *Veillonella dispar,* and *Pseudomonas aeruginosa*. Total DNA was extracted and prepared for sequencing using KAPA HyperPlus (KAPA Biosystems, Wilmington, MA). The libraries were sequenced on an Illumina MiSeq platform using a Reagent Kit v3 150-cycle (Illumina, San Diego, CA, USA). Quality filtering, dereplication and cloning vector removal were performed as above. Sequences were assembled using Spades [65]. Genome completeness was estimated in ANVI’O [66] through a HMM profile search of bacterial single-copy core genes. The coverage of each of these genomes in the metagenomes and transcriptomes varied from 0 to 851 per sample, as shown in Figure 1. L50 varied from 9.4 Kb (*Streptococcus*) to 1456 Kb (*Rothia*). The closest relatives of each isolate were obtained through the Similar Genome Finder Tool in the Pathosystems Resource Integration Center (PATRIC), which searches all public genomes using Mash, a k-mer based tool for whole-genome distance pairwise estimation (Table S2) [67].

Metabolomics: Sputum samples (1 mL) were thawed and treated with 40 µL 20% dihidroteitrol (DTT dissolved in water), followed by vortexing. Samples were frozen at −80 °C, then lyophilized before extraction to concentrate all metabolites. A methylchloroformate (MCF) derivatization was used as described by Smart et al., 2010 [68]. Briefly, after lyophilization, 150 µL 2N NaOH were added to each sample and vortexed. Then, 150 µL 100% methanol were added, followed by 150 µL 10 mM L-methionine (2,3,3,4,4-D5; methyl-D3), as internal standard (Cambridge Isotope Laboratories, Tewksbury, MA, USA) and 20 µL pyridine as a catalyst. For derivatization, 40 µL methyl chloroformate (MCF) were added, followed by vortexing 30 s. Another 40 µL MCF were added, with 30 s of vortexing. Samples were extracted using chloroform, dried over sodium sulfate, and analyzed directly by gas chromatography–mass spectrometry (GC-MS). The metabolites were identified using the NIST library (2007) using the Automated Mass Deconvolution and Identification System (AMDIS) program. 

Metatranscriptomics: Total RNA was extracted from raw sputum samples using guanidinium thiocyanate according to manufacturer’s protocol (TRIzol, Invitrogen, Waltham, MA, USA). RNA was resuspended in 50 µL of RNAse-free water and DNase treatment was performed by adding 2 µL of TURBO DNase (Thermo Fischer Scientific, Waltham, MA, USA). First strand RNA was synthesized using Superscript II reverse transcriptase (Invitrogen, Waltham, MA, USA). Sequencing libraries were prepared using the Ovation Complete Prokaryotic RNA-Seq System (NuGEN Technologies, Redwood City, CA, USA). Libraries were sequenced using a Reagent Kit v3 150-cycle in a MiSeq platform (Day 0) and NovaSeq platform (all other samples, 20M reads per sample) (Illumina, San Diego, CA, USA). Quality filtering, dereplication and cloning vector removal were performed as above (Table S1, Supplementary Data S1). The transcripts were mapped to the NCBI RefSeq database of complete bacterial genomes using SMALT at 96% identity. We also mapped the transcripts to the genomes of bacterial isolates obtained from the same patient at 96% identity using SMALT.

Quantitative metagenomics and transcriptomics: The interpretation of omics datasets is largely limited by the analyses of relative abundances that prevent accurate between-sample comparisons [50]. In these datasets, the relative abundance of one community member can increase or decrease even when its actual abundance in the original sample remained the same due to variations in abundance of other community members. Obtaining actual microbial abundances and transcriptional activities per ml of sputum was essential to accurately inform antibiotic treatment decision in the present study. To solve this problem, we developed a quantitative metagenomics and transcriptomics approach integrating epifluorescence microscopy counts of the total microbial abundance, complete genomes, and metagenomic and transcriptomic sequence data. For metagenomes, the number of reads mapping to the bacterial genomes sequenced from clinical isolates (as shown earlier) was normalized by genome length using FRAP [69] to calculate the relative abundances at species level in the metagenome. The relative abundances were multiplied by the total microbial abundances determined by epifluorescence microscopy, yielding the number of cells of each species per ml of sputum. A similar approach was utilized for metatranscriptomes, where the number of transcripts mapping to a genome was normalized by genome size using FRAP [69] to calculate relative number of transcripts per taxon per sample. This relative transcriptional activity was multiplied by the number of cells per ml of sputum to obtain the taxon-specific transcriptional activity per ml of sputum. The relative transcript abundance was normalized by microbial abundances per mL of sample to allow between-sample comparisons. This scaling assumes that the total bacterial transcriptional activity per ml of sputum is proportional to the bacterial abundance per ml of sputum. The transcript recruitment to the genomes of clinical isolates was visualized using ANVI’O [66].

Multivariate analyses: Microbial abundances were combined with taxon-specific transcriptomic activity per mL, WinCF fermentation, and total microbial abundances in one dataset (Supplementary Data S1–S3) and analyzed through a non-metric multidimensional scaling (NMDS) using Bray-Curtis distances in the vegan package in R. The two first dimensions are visualized in Figure 1a. Samples were clustered using the proximity matrix from an unsupervised random forest in the R package randomForest using 5000 trees and Ward method for hierarchical clustering. Random forest is a robust non-parametric statistical learning method for the identification of clusters and variables of importance in complex multi-omics data [70,71]. The effects of different antibiotic regimes (clindamycin, no antibiotics, and the combination of colistin, piperacillin-tazobactam, ceftazidime-avibactam, and vancomycin) were tested using a supervised permutational random Forest (5000 trees and 1000 permutations) where the three antibiotic regimes were used as supervising variable in the package rfPermute.

## 5. Conclusions

The integration of omics tools presented here demonstrates in vivo the role of facultative anaerobes, mainly *Streptococcus*, as keystone bacteria in a CF microbiome. These anaerobes release fermentation products that sustain the growth of the classic pathogen *P. aeruginosa*. Disturbing community stability with antibiotics targeting Gram-positive and anaerobic bacteria improves patient lung function. By applying the ecological concept of keystone species, this study points toward alternative antimicrobial treatments for polymicrobial diseases guided by personalized multi-omic analyses.

## Data Availability

Metagenomic data is available in the NCBI Short Read Archive (SRA) under BioProject ID PRJNA580503. Genome sequences of clinical isolates are provided as contigs and associated annotation files in the Pathosystems Resource Integration Center (PATRIC) server in the public workspace csilveira/CF146. Transcriptomic data are available through the Gene Expression Omnibus (GEO) server in NCBI record GSE139943. Codes used for statistical analysis are provided in github under cbsilveira/CF146 and csv files used as input are provided as Supplemental Material along with the manuscript.

## Supplementary Materials

The following are available online at https://www.mdpi.com/article/10.3390/ijms222112050/s1.
