# Supplementary material for: Multi-Omics Study of Keystone Species in a Cystic Fibrosis Microbiome
Source: Int J Mol Sci. 2021 Nov 7;22(21):12050. doi: 10.3390/ijms222112050 (PMC8584531; doi:10.3390/ijms222112050)
Supplement: Supplementary file 1 [file ijms-22-12050-s001.zip › Supplemental.pdf]

Figure S1

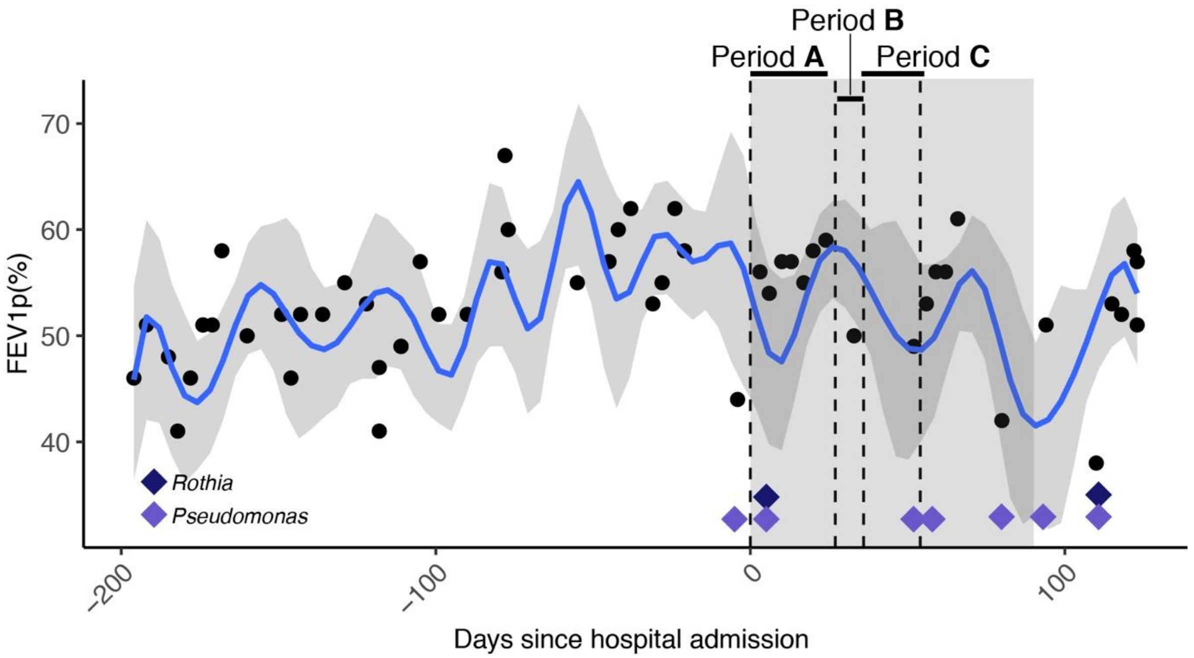

Table S1

Day	Type	Raw reads	Post QC	% passing QC	Post UniVec	% Post UniVec	Post Human	% Non-human	Post rRna	% non-rRNA
16	Transcriptome	18074302	17975310	99.45	17974832	100.00	4252450	23.65	302166	7.10
21	Transcriptome	17502515	17278885	98.72	17278462	100.00	9518790	55.09	1956673	20.55
28	Transcriptome	14879699	11673957	78.46	11673596	100.00	1571497	13.46	645395	41.06
30	Transcriptome	16431639	16377739	99.67	16377267	100.00	1043638	6.37	284029	27.21
33	Transcriptome	22280383	21975912	98.63	21975146	100.00	4327858	19.69	953549	22.03
35	Transcriptome	21560478	21139180	98.05	21138779	100.00	1476280	6.98	1057092	71.60
37	Transcriptome	14187420	14042449	98.98	14039450	99.98	3533234	25.16	523023	14.80
40	Transcriptome	17444562	16285279	93.35	16277563	99.95	9144485	56.17	1322207	14.45
0	Metagenome	2100408	2095156	99.75	2095156	100.00	68654	3.27		
16	Metagenome	2844204	2838884	99.81	2838884	100.00	40383	1.42		
21	Metagenome	2364230	2360358	99.84	2360358	100.00	192992	8.17		
28	Metagenome	2100408	2096475	99.81	2096475	100.00	10400	0.49		
30	Metagenome	1613441	1610522	99.82	1610522	100.00	9155	0.56		
33	Metagenome	1870463	1864329	99.67	1864329	100.00	27902	1.49		

35	Metagenome	2017235	2013837	99.83	2013837	100.00	9135	0.45
37	Metagenome	1454998	1452624	99.84	1452624	100.00	7305	0.50
40	Metagenome	1564910	1561774	99.80	1561774	100.00	33185	2.12

Table S2

**Clinical Isolate**	**genome length**	**GC content**	**Completeness**	**Closest genome**	**Distance**
Pseudomonas aeruginosa CF146	6652278	66.1	99.30	*Pseudomonas aeruginosa* strain 2671	0.0012
Staphylococcus haemolyticus CF146	2476233	32.63	100.00	*Staphylococcus haemolyticus* JCSC1435	0.0065
Rothia mucilaginosa CF146	2219659	61.9	98.60	*Rothia mucilaginosa* strain 473_RMUC	0.0096
Streptococcus sanguinis CF146	3339201	40.62	94.00	*Streptococcus sanguinis* strain NCTC3168	0.0539
Veillonella dispar CF146	2105769	38.82	93.00	*Veillonella dispar* strain MGYG-HGUT-00214	0.0816
